# Oral application of vancomycin alters murine lung microbiome and pulmonary immune responses

**DOI:** 10.1002/iid3.675

**Published:** 2022-07-14

**Authors:** Stefan Pfeiffer, Gregor Jatzlauk, Joni V. Lund, Eistine Boateng, Draginja Kovacevic, Machteld N. Hylkema, Sabine Bartel, Michael Schloter, Susanne Krauss‐Etschmann

**Affiliations:** ^1^ ZIEL—Institute for Food and Health Technical University of Munich Freising Germany; ^2^ Department for Environmental Health Research Unit for Comparative Microbiome Analysis, Helmholtz Zentrum München Neuherberg Germany; ^3^ Division of Early Life Origins of Chronic Lung Diseases, Research Center Borstel, Leibniz Lung Center, Airway Research Center North (ARCN) German Center for Lung Research (DZL) Borstel Germany; ^4^ DZL Laboratory for Experimental Microbiome Research, Research Center Borstel, Leibniz Lung Center, Airway Research Center North (ARCN) German Center for Lung Research (DZL) Borstel Germany; ^5^ Department of Pathology and Medical Biology University of Groningen, University Medical Center Groningen Groningen The Netherlands; ^6^ GRIAC Research Institute University of Groningen, University Medical Center Groningen Groningen The Netherlands; ^7^ Institute for Experimental Medicine Christian‐Albrechts‐Universität zu Kiel Kiel Germany

**Keywords:** early life antibiotics, gut microbiome, gut–lung axis, lung inflammation, lung microbiome

## Abstract

Early life exposures to antibiotics negatively impact respiratory health and are associated with an increased risk of childhood asthma. It is explained that the lung is inclined to develop chronic inflammatory phenotypes due to early antibiotic alteration in the gut microbiome. We investigated whether a gut‐targeted antibiotic has an impact on the lung microbiome and on pulmonary immunity. Fourteen‐day old C57BL/6 mice were administered with vancomycin via oral gavage for 3 days (1 time/day). Control groups were treated with clarithromycin and phosphate‐buffered saline (PBS), respectively. Five days after treatment, the cecum and lung microbiome, and pulmonary immune response were analyzed. Vancomycin treatment decreased the relative abundance of the genera *Clostridium* XIVa and *Alistipes* and the family *Lachnospiraceae* in the cecum. Furthermore, the relative abundance of the family *Parabacteroidetes* and the genus *Lactobacillus* were increased, whereas the abundance of the phylum Firmicutes was decreased. In the lung, vancomycin treatment reduced bacteria belonging to *Clostridium XIVa* and the family *Lachnospiraceae* as compared to those in the clarithromycin treated group. Lung cells from the vancomycin‐treated mice released higher levels of interleukin (IL)‐4 and IL‐13 compared to those from the PBS group, and increased levels of IL‐6, IFN‐γ, and TNFα compared to lung cells from the clarithromycin and PBS treated mice. Our pilot study suggests that alteration in the gut microbiome could affect bacterial composition and immunity of the lung hence proposes a gut–lung microbiome axis in early life.

## Graphical abstract

Early life exposures to antibiotics negatively impact respiratory health and are associated with an increased risk of childhood asthma. We investigated whether a gut‐targeted antibiotic has an impact on the lung microbiome and on pulmonary immunity. Our pilot study suggests that alteration in the gut microbiome could affect bacterial composition and immunity of the lung hence proposing a gut–lung microbiome axis in early life.

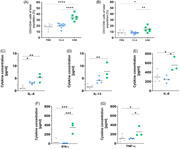

**Abbreviations**
BALF: bronchoalveolar lavage fluid
CLA: clarithromycin
IFN‐γ: interferon‐gamma
IL: interleukin
OVA: ovalbumin
PBS: phosphate‐buffered saline
Th2: T helper cell type 2
TNF‐α: tumor necrosis factor alpha
VAN: vancomycin

## 1 INTRODUCTION

A repertoire of research studies associates exposure to antibiotics in early life with the development of allergy and asthma in childhood. Antibiotics contribute to fluctuations in the diversity of microbial communities in the host. It is discussed that the gut microbiome undergoes dynamic changes in the first 3 years of life until a more stable and diverse community is established.^1^ This microbial colonization is suggested to influence the maturation and functional development of immune cells.^2^, ^3^ These reports potentially underscore the notion that intermittent modulation of the gut microbiome with antibiotics in early life could be detrimental to the immune system. In line with this, an earlier study demonstrated that oral vancomycin (VAN) use during pregnancy followed by early postnatal treatment reduced the gut microbial diversity in infant mice, and further aggravated the severity of ovalbumin (OVA)‐induced experimental asthma.^4^, ^5^ Indeed, there is evidence that dysbiosis in the gut microbiome is a trigger for respiratory diseases. To add to what is already known, we investigated if the gut–lung microbiome axis^6^ prevails in early life and influences immune cell response. We treated mice with VAN, an antibiotic not absorbed in the gut, or clarithromycin (CLA), a macrolide that is resorbed from the gut for systemic distribution. In general, we proposed that a gut–lung microbiome axis in infants could be one contributing factor reinforcing risks to chronic inflammatory diseases later in life.

## 2 RESULTS AND DISCUSSION

**Figure 1:**
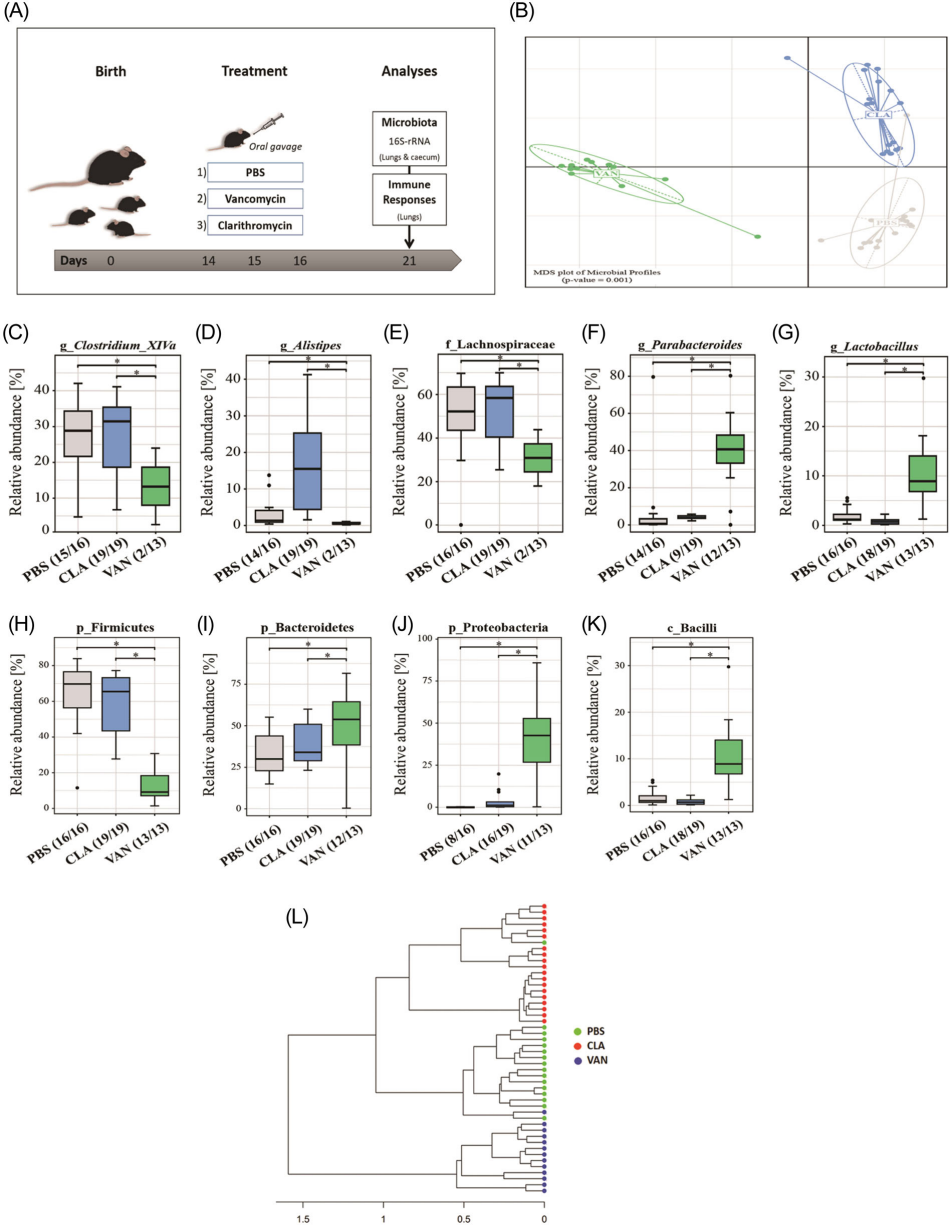
(A) Overview of experimental model. Analysis of gut microbiota (cecal samples): (B) Unconstrained nMDS plot of generalized UniFrac distances; (C–K) impact of antibiotics on particular bacterial taxa. (L) 16S dendrogram (Ward's clustering method) based on generalized UNiFrac distances. PBS (*n* = 16), CLA (*n* = 19), and VAN (*n* = 13). g (genus), f (family), p (phylum), c (class). *p < .05 indicates significant differences. CLA, clarithromycin; nMDS, nonmetric multidimensional scaling; PBS, phosphate‐buffered saline; VAN, vancomycin.

In this study, we applied the locally acting VAN via oral gavage to 14‐day‐old C57BL/6 male and female mice for 3 days and analyzed the gut and lung microbiome as well as pulmonary immune cells 5 days after the last antibiotic treatment (Figure 1A). As controls, we treated one group of infant mice with CLA and another group with phosphate‐buffered saline (PBS). Care was taken to avoid gavage‐related refluxes and esophageal trauma during orogastric application of VAN, CLA, and PBS.

As expected, compared to PBS, both VAN and CLA administration significantly changed bacterial communities in murine ceca with regard to β‐diversity (Figure 1B) and their richness (Supporting Information: Figure S1A), although VAN showed a stronger decrease in bacterial richness than CLA. Further, we assessed the impact of the antibiotics on bacterial genera (Supporting Information: Figure S2A) and made a compilation of the top‐ranked bacteria (Supporting Information: Table 1) in response to the treatments. Compared to both control groups, VAN significantly reduced the relative abundance of bacteria belonging to the genera *Clostridium* XIVa and *Alistipes* as well as the family *Lachnospiraceae*, whereas the relative abundance of the family *Parabacteroidetes* and the genus *Lactobacillus* were significantly increased in the cecum (Figure 1C–G). To confirm our observations, we analyzed the impact of antibiotic treatments on the phylum level. Compared to PBS and CLA, VAN significantly reduced the relative abundance of the phyla Firmicutes (Figure 1H), under which the genus *Clostridium* XIVa and the family *Lachnospiraceae* are classified. In contrast, the phyla Bacteroidetes and Proteobacteria, and the class Bacilli, followed opposite trends (Figure 1I–K). Moreover, Figure 1L shows a 16S dendrogram describing the phylogenetic distance between samples in terms of relatedness and abundance.

**Figure 2:**
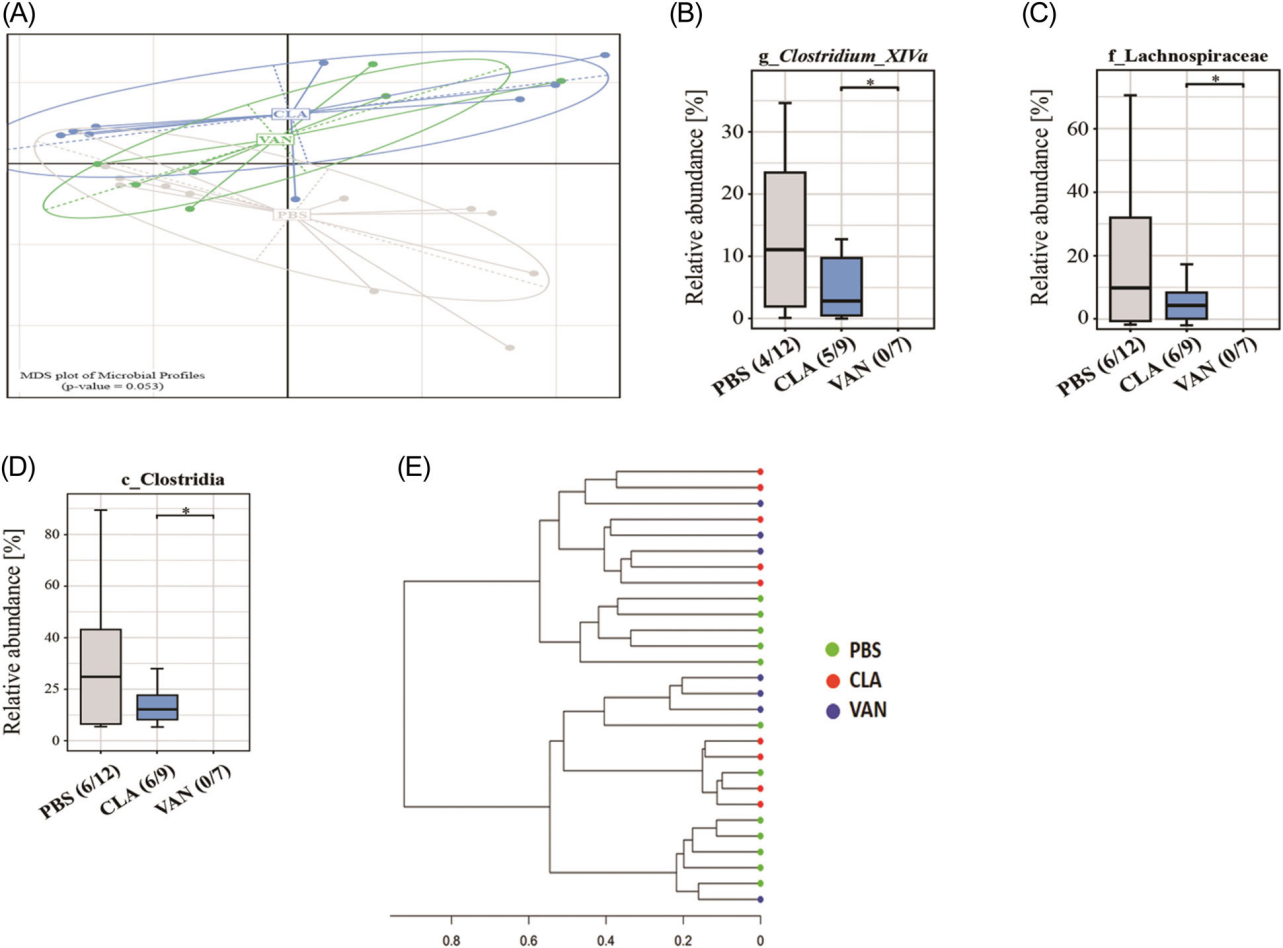
Analysis of lung microbiota. (A) Unconstrained nMDS plot of generalized UniFrac distances; (B–D) impact of antibiotics on some specific lung bacterial taxa. (E) 16S dendrogram (Ward's clustering method) based on generalized UNiFrac distances. PBS (*n* = 12), CLA (*n* = 9), and VAN (*n* = 7). Numbers in brackets below the graphs show the number of samples within which a certain taxon was detected (e.g., PBS [4/12] means the taxon was detected in 4 samples out of 12). *p < .05 indicates significant differences. CLA, clarithromycin; nMDS, nonmetric multidimensional scaling; PBS, phosphate‐buffered saline; VAN, vancomycin.

VAN is not absorbed in the gut, and for this reason, we assessed whether the antibiotic could alter the lung microbiome. We speculated that changes in the lung microbiome may trigger cellular responses which could influence respiratory health in accordance with the findings by Russell et al. Surprisingly, while there was no change in bacterial richness in the lung (Supporting Information: Figure S3A), the beta diversity analyses (Figure 2A) followed the same trend as observed in the cecum (Figure 1B). Furthermore, based on the top‐ranked bacteria in the murine lung (Supporting Information: Figure S2B and Table S2) VAN treatment induced a significant decrease in bacteria belonging to *Clostridium XIVa* and the family *Lachnospiraceae* as compared to CLA‐treated mice (Figures 2B,C). Additionally, the relative abundance of Clostridia was significantly reduced in VAN‐treated mice compared to those in the CLA group only (Figure 2D). The phylogenetic distance between samples in terms of relatedness and abundance is indicated with a 16S dendrogram in Figure 2E.

**Figure 3:**
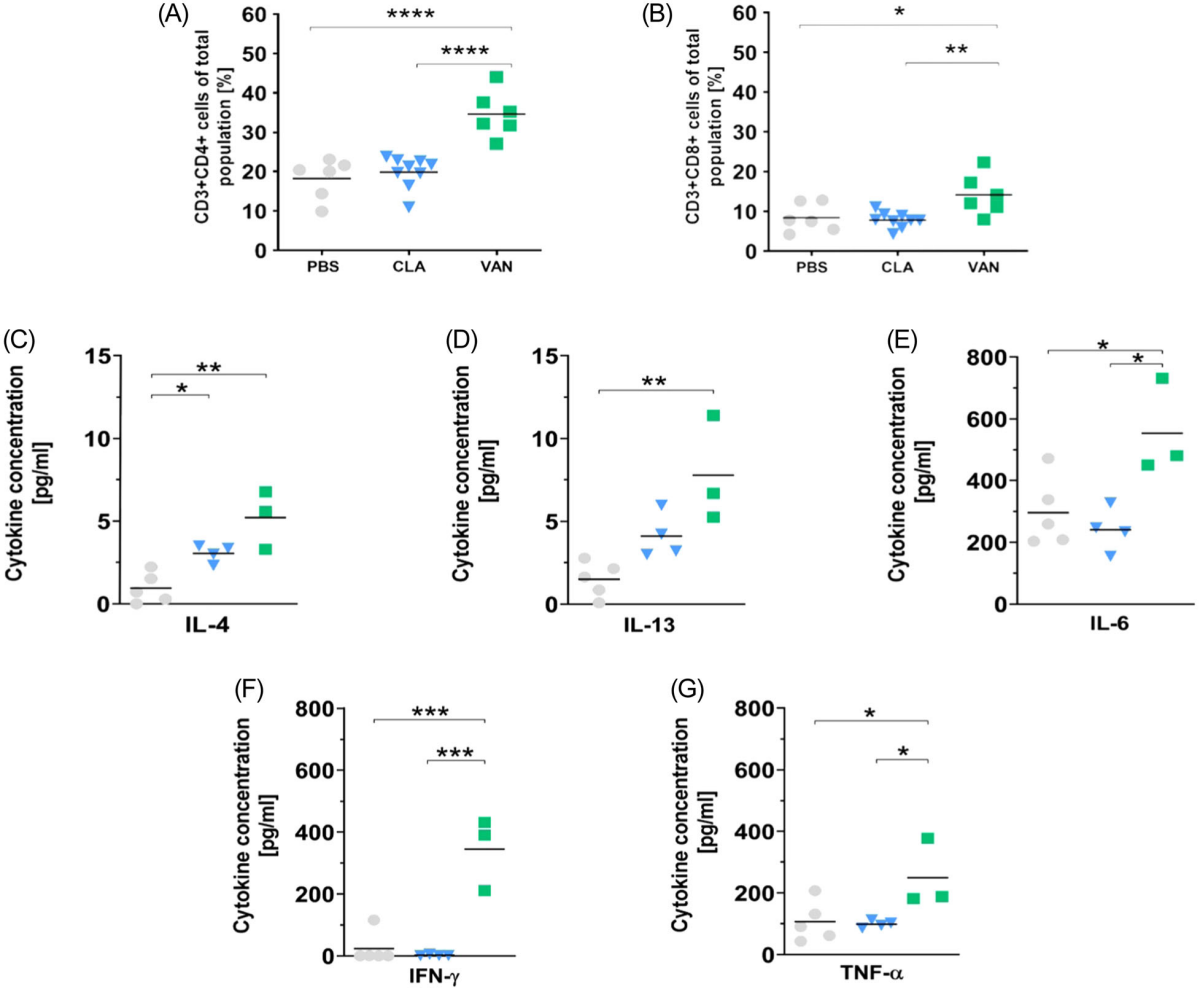
(A, B) T cell population in the lungs. n for PBS, CLA, and VAN are 6, 9, and 6, respectively. (C–G) cytokines in the supernatant of CD3/CD28 stimulated cell cultures of lung cells (The scales differ according to the variable abundance of the cytokines). *n* = 5 (PBS), *n* = 4 (CLA), *n* = 3 (VAN), and each *n* represents pooled samples. *p < .05, **p < .01, ***p < .001, and ****p < .0001 represent significant differences. CLA, clarithromycin; PBS, phosphate‐buffered saline; VAN, vancomycin.

We next assessed whether the observed changes in bacterial diversity correspond with immune modulation in the lung. To do this, total cell count in bronchoalveolar lavage fluid (BALF) and T cell populations in single‐cell suspensions from the lungs were analyzed. Using the flow cytometry gating strategy in Supporting Information: Figure S4, we noticed a remarkable increase in CD4+ T cells (Figure 3A) and a slight increase in CD8+ T cells (Figure 3B) in the VAN group compared to the CLA and PBS treated mice. Further, we cultured cells isolated from the lungs and stimulated them via CD3/CD28 to investigate the levels of some selected cytokines released by the cells (Figure 3 and Supporting Information: Figure S5). The present data showed an increase in Th2‐associated cytokines interleukin (IL)‐4 and IL‐13 in cells isolated from the VAN‐treated mice compared to those from the PBS‐administered mice (Figure 3C,D). Significant amounts of pro‐inflammatory IL‐6, IFN‐γ, and TNFα, were also released by the cells from the VAN group compared to those isolated from the CLA and PBS groups (Figure 3E–G). Overall, our findings implicate an alteration of the pulmonary immune development/responses in the lungs of mice treated with VAN in early life. This is characterized by a mixed pro‐inflammatory phenotype shown by increased T cell counts and not only Th1 but surprisingly common Th2 cytokines (IL‐4, IL‐13). These changes in immune response may support the idea that an antibiotic‐induced shift in the gut microbiota could provoke Th2 hyperreactivity to trigger the development of atopic diseases later in life.

## 3 CONCLUDING REMARKS

In brief, orally administered VAN acts locally in the gut, however, simultaneous trends in the lung microbial community probably suggest systemic adaptability to treatment. We understand that the translocation of bacteria or bacterial fragments,^7^ release of bacterial metabolites, for example, short‐chain fatty acids,^8^ and the trafficking of immune cells from the gut to the lung^9^ are possible explanations for the observations in our study. Moreover, we speculate a gut–lung axis to that effect. In addition to what was previously explained,^6^ here, the gut microbial changes may have induced moderate alterations in the lung environment or comparatively, showed similar trends as observed in the lung—pointing to a possible gut–lung–microbiome axis. This raises the question of the extent to which microbes at different body sites connect with each other and how a local antibiotic treatment could affect other microbial communities. Further studies are therefore needed for sufficient evidence and clarity on the concept before probing into the mechanistic aspect. Taken together, our pilot study suggests that changes in the gut microbiome could have consequences on the bacterial composition and immunity of the lung and this may contribute to the reported increase in asthma and allergies in individuals given antibiotics in the first years of life.

## AUTHOR CONTRIBUTIONS

*Conceptualization*: Susanne Krauss‐Etschmann, Sabine Bartel, and Michael Schloter. *Methodology*: Stefan Pfeiffer and Gregor Jatzlauk. *Formal analysis and investigation*: Stefan Pfeiffer, Gregor Jatzlauk, Joni V. Lund, and Draginja Kovacevic; *Resources*: Susanne Krauss‐Etschmann. *Data curation*: Gregor Jatzlauk, Joni V. Lund, Draginja Kovacevic, Machteld N. Hylkema, Michael Schloter, and Susanne Krauss‐Etschmann. *Writing—original draft preparation*: Susanne Krauss‐Etschmann, Stefan Pfeiffer, Gregor Jatzlauk, Joni V. Lund, and Eistine Boateng. *Writing—review and editing*: Susanne Krauss‐Etschmann, Stefan Pfeiffer, Gregor Jatzlauk, Joni V. Lund, Eistine Boateng, Draginja Kovacevic, Machteld N. Hylkema, Sabine Bartel, and Michael Schloter. *Visualization*: Draginja Kovacevic. *Supervision*: Susanne Krauss‐Etschmann and Michael Schloter. *Funding acquisition*: Susanne Krauss‐Etschmann and Michael Schloter. All authors have read and agreed to the published version of the manuscript.

## CONFLICTS OF INTEREST

The authors declare no conflicts of interest except for Sabine Bartel who reports grants and personal fees from Bencard Allergie GmbH which had no role in the design of the study; in the collection, analyses, or interpretation of data; in the writing of the manuscript, or in the decision to publish the results.

## ETHICS STATEMENT

The animal study protocol was approved by the Institutional Review Board of the Government of the District of Schleswig—Holstein (V 244—14538/2016 (10‐1/16).

## Supporting information

Supporting information.

## Data Availability

Sequences are currently in the process of submission to the NCBI Sequence Read Archive.

## References

[1] Stewart CJ , Ajami NJ , O'Brien JL , et al. Temporal development of the gut microbiome in early childhood from the teddy study. Nature 2018;562:583‐588. 30356187 10.1038/s41586-018-0617-x PMC6415775

[2] Dzidic M , Boix‐Amoros A , Selma‐Royo M , Mira A , Collado MC . Gut microbiota and mucosal immunity in the neonate. Med Sci (Basel). 2018;6(3):56. 10.3390/medsci6030056 PMC6163169 30018263

[3] Geuking MB , Cahenzli J , Lawson MA , et al. Intestinal bacterial colonization induces mutualistic regulatory T cell responses. Immunity. 2011;34:794‐806. 21596591 10.1016/j.immuni.2011.03.021

[4] Russell SL , Gold MJ , Hartmann M , et al. Early life antibiotic‐driven changes in microbiota enhance susceptibility to allergic asthma. EMBO Rep. 2012;13:440‐447. 22422004 10.1038/embor.2012.32 PMC3343350

[5] Man WH , Clerc M , de Steenhuijsen Piters WAA , et al. Loss of microbial topography between oral and nasopharyngeal microbiota and development of respiratory infections early in life. Am J Respir Crit Care Med. 2019;200:760‐770. 30883192 10.1164/rccm.201810-1993OC

[6] Dang AT , Marsland BJ . Microbes, metabolites, and the gut‐lung axis. Mucosal Immunol. 2019;12:843‐850. 30976087 10.1038/s41385-019-0160-6

[7] Wang H , Zhang W , Zuo L , et al. Intestinal dysbacteriosis contributes to decreased intestinal mucosal barrier function and increased bacterial translocation. Lett Appl Microbiol. 2014;58:384‐392. 24354719 10.1111/lam.12201

[8] Trompette A , Gollwitzer ES , Pattaroni C , et al. Dietary fiber confers protection against flu by shaping Ly6c(‐) patrolling monocyte hematopoiesis and Cd8(+) T cell metabolism. Immunity. 2018;48:992‐1005.e8. 29768180 10.1016/j.immuni.2018.04.022

[9] Russell SL , Gold MJ , Willing BP , Thorson L , McNagny KM , Finlay BB . Perinatal antibiotic treatment affects murine microbiota, immune responses and allergic asthma. Gut Microbes. 2013;4:158‐164. 23333861 10.4161/gmic.23567 PMC3595077

