# Supplementary material for: Oral application of vancomycin alters murine lung microbiome and pulmonary immune responses
Source: Immun Inflamm Dis. 2022 Jul 14;10(8):e675. doi: 10.1002/iid3.675 (PMC9281482; doi:10.1002/iid3.675)
Supplement: Supplementary file 1 — Supporting information. [file IID3-10-e675-s001.docx]

**Supporting Information**

**Materials and methods**

**Animals**

All animal studies were performed according to the German law of animal welfare and were approved by the Government of the District of Schleswig - Holstein (V 244 – 14538/2016 (10-1/16). C57BL/6 mice were housed in individually ventilated cages (Sealsafe PLUS, Green Line, Tecniplast, Italy) under specific pathogen-free conditions, on a 12-hour light-dark cycle with access to standard food and water *ad libidum*.

7-9-week-old males and females (Charles River GmbH, Sulzfeld, Germany) were mated at a ratio of 1:2 for 48 h. Pregnant females were fed with breeding chow (Maus-Zucht and Energie-reichs, sniff-Spezialitäten GmbH, Soest, Germany). Littermates were nursed by the mother until sacrificed for sample collection. At the age of 14 days, offspring received either 30 mg/kg VAN (Hikma Farmaceutica, Terrugem, Portugal) or 15 mg/kg CLA (Hexal AG, Holzkirchen, Germany) dissolved in sterile PBS (100 µl/ 10 g body weight), on three consecutive days via oral gavage (Figure 1A). Control mice received PBS only. Animals were sacrificed in deep anaesthesia 5 days after the last antibiotic treatment.

**Tissue collection**

After sacrificing the mice, lungs were used either for immune or microbiome analysis. For immune analysis, bronchoalveolar lavage (BAL) was collected by rinsing the lungs with 500 µl cold PBS via a tube inserted in the trachea. Lungs were then perfused via the heart with 5 ml PBS and immediately processed for cell culture and flow cytometric analysis. For microbiome analysis, lungs and caecum (representing gut) were collected with sterilized instruments and stored at -80 °C until further analysis.

## DNA extraction

All steps for DNA extraction, PCR, and amplicon library preparation were performed in biosafety cabinets. The frozen caeca were weighed and homogenized in 400 µl ddH_2_0 using a Precellys24 homogenizer (Bertin Technologies, Montigny le Bretoneux, France) at 5.5 m/s for 30 s. DNA was extracted out of 100 µl of the homogenized tissue using the QIAamp PowerFecal DNA Kit (Qiagen, Venlo, NL) following the manufacturer’s recommendations. Frozen lung samples were likewise weighed and homogenized. 180 µl lysozyme solution (20 mg/ml) (Hoffmann - LaRoche, Basel, Switzerland) was added to 200 µl tissue homogenate and the mixture was incubated at 37°C for 30 minutes under slow agitation. Further steps were conducted according to the manufacturer’s protocol for Gram-positive bacteria provided by the PureLink™ Genomic DNA Mini Kit (ThermoFisher Scientific, Altham, USA).

## 16S rRNA gene amplification and high throughput sequencing

For both caecum and lung samples, we used the primer pair 347F / 803R, which amplifies the hypervariable regions V3-4 of the 16S rRNA gene [7]. PCRs were performed in triplicates to reduce common PCR bias and using the hot-start technique to reduce the probability of dimer formation. PCR conditions were as follows: Initial denaturation 98°C / 30 s; 33 cycles for lung samples and 20 cycles for caecum samples (denaturation 98°C / 30 s, annealing 58°C /30 s, elongation at 72°C / 30 s; final elongation 72°C / 5 m). In addition to the samples, we included NE controls as well as PCR negative controls (NTC). We further included for the lung samples one NE and NTC control that was spiked with 10 ng of the commercial bacterial mock community HM-783D (BEI Resources, NIAID, NIH, USA) to estimate the contamination risks and chimeric biases that could be introduced due to the high number of amplification cycles. Success of each of the PCRs was veriﬁed with agarose gel electrophoresis. Pooled PCR triplicates were puriﬁed with Agencourt AMPure XP paramagnetic beads (Beckman Coulter, Brea, USA). Purified PCR products were checked for dimers and were quantified using the DNF-473 Standard Sensitivity NGS Fragment Analysis Kit on the Fragment Analyzer (Advanced Analytical, Ankeny, USA). The library preparation was conducted according to the Illumina guidelines for 16S rDNA gene amplicon preparation with modifications.

25µl Indexing PCR reactions contained 2.5 µl of forward and reverse indexing primers (Nextera® XT Index Kits Illumina, Inc., CA, USA), 12.5 µl 2X PCR Master Mix and 10 ng of the NEBNext® High-Fidelity 2X PCR Master Mix (New England Biolabs Inc., MA, USA). Indexing PCR conditions were as follows: 30 s of initial denaturation at 98°C; 8 cycles including denaturation at 98°C for 30 s, 30 s annealing at 55°C and 30 s elongation at 72°C; a final 5 min elongation step at 72°C. Verification of PCR, purification and quantification of PCR products was conducted as described for the amplicon PCR. PCR products were diluted to 4 nmol/L and pooled. For sequencing, 8 pM of DNA spiked with 20 % PhiX were loaded and the paired-end sequencing was performed on a MiSeq® System (Illumina, Inc., CA, USA) using the MiSeq® Reagent Kit v3 (600 cycle) for paired-end sequencing according to the manufacturer’s guidelines.

**Preparation of lung cells suspension**

The whole lung was cut into small pieces and digested for 45 min at 37 °C with collagenase diluted in PBS (0.5 mg/ml, Sigma-Aldrich Chemie GmbH, Steinheim, Germany) under sterile conditions. The suspension was homogenized by repeated agitation through a long cannula and afterwards filtered using a 70 µm cell strainer. Next, red blood cells were lysed by Gey´s lysis buffer (10 mM KHCO_3_ Merck, 155 mM NH_4_Cl Merck, 100 µM EDTA Sigma in aqua dest.) for 1 min, and washed twice with PBS. A sample of the single cell suspension (end volume 5 ml) was mixed with trypan blue (1:2) and counted using a hemocytometer.

**Cytokines analysis**

1*10^6^ lung cells were suspended in 500 µl TexMACS medium (Miltenyi Biotec GmbH, Bergisch- Gladbach, Germany) supplemented with 0.01 mM 2-Mercaptoethanol (Miltenyi), 10 % FCS (Biochrom AG, Berlin, Germany) and 100 IU Penicillin/Streptomycin (Biochrom) and stimulated with anti-CD3/CD28 MACS beads (T Cell Activation/Expansions Kit Mouse, Miltenyi Biotec) overnight at 37 °C and 10% pCO_2_. Cell culture supernatant was collected (300x g, 10 min, 21 °C) and frozen at -80 °C until cytokine analysis. The BD cytometric bead array (CBA) Enhanced Sensitivity Flex Set System for mouse (all Becton Dickinson GmbH, Heidelberg, Germany) was used to measure IL-1β, IL-4, IL-5, IL-6, IL-10, IL-12p70, IL-13, IL-17A, IFN-γ and TNFα in the cell culture supernatant. The analysis was performed according to the manufacturer´s instructions except capture and detection antibodies were pre-diluted at 1:2. MACSquant10 (Miltenyi) was used for the measurements.

**Flow cytometry**

1*10^6^ lung cells of the single cell suspension were analysed to differentiate the T-cell populations by flow cytometry using the following antibodies: α-mouse CD3e-APC 1:100 (Miltenyi Biotec, Bergisch Gladbach, Germany, clone REA641, 30 µg/ml), α-mouse CD4-APC-H7 1:600 (Becton Dickinson Biosciences, Franklin Lakes, USA, clone GK1.5, 200 µg/ml) and α-mouse CD8b-eFlour 450 1:400 (eBioscience Affymetrics, San Diego, USA, clone eBioH35-17.2, 500 µg/ml). Annexin-V-FITC 1:50 (eBioscience Affymetrics) and Fixable Viability Dye eFluor 455UV 1:1000 (eBioscience Thermo Fisher, Waltham, USA) were used to identify living cells. Fc-receptors were blocked with Fc-Block (1:50, BD, clone 2.4G2, 500 µg/ml) in PBS for 10 min at 4°C. Then, antibody mixture was added and incubated for 15 min, at room temperature (RT). After washing twice with PBS, the cells were incubated with Fixable Viability Dye for 15 min (RT, dark) followed by Annexin-V staining (Annexin V Apoptosis Detection Kit, eBioscience Affymetrics). After washing, cells were fixed with 4% paraformaldehyde (Fluka Honeywell, Seelze, Germany) and finally suspended in PBS with 10% foetal calf serum (Biochrom, Berlin, Germany). The analysis was done on a BD LSR II using the software FACSDiva 8 (both Becton Dickinson).

## Bioinformatic analysis

Amplicons were processed using established protocols (https://doi.org/10.5281/zenodo.1302799) based on USEARCH v.10.2.240 and VSEARCH [9]. In short, USEARCH and VSEARCH were used to merge demultiplexed raw reads in a single file, remove primers and to perform quality filtering with maxee 1.0 as a threshold. Denoising was performed using the Unoise3 algorithm to identify sequence variants (zero radius OTUs = zOTUs) with a minimum length of 350 bp. The resulting zOTU table was reanalysed using the UNCROSS algorithm to remove sequencing errors based on wrong barcoding assignment through cross-feed [10]. Taxonomy was predicted using RDP reference database v16 [11] using the SINTAX algorithm [12] with a conﬁdence cut-off at 0.5. A Newick taxonomic tree was generated with the cluster –agg command in USEARCH. Nonprokaryotic zOTUs (eukaryotes, chloroplast, and mitochondria) and singletons were removed. zOTUs that could be detected consistently in NE and NTC controls were removed, as recommended [13]. Further, comparison of samples and controls spiked with the HMD783 mock community showed that the level of contamination was neglectable.

## Statistical analysis

To perform statistical analysis of the sequencing data in R, we applied FASPA-scripts (https://doi.org/10.5281/zenodo.1302799) to enable downstream analysis using the R-packages phyloseq [14], the Rhea script collection [15], vegan (www.pelagicos.net/MARS6910_spring2015/manuals/R_vegan.pdf). Normalization to 5000 zOTUs/sample, calculation of α-diversity metrices (Observed Species Richness, Shannon diversity), calculation of β-diversity unconstrained nonmetric multidimensional scaling (nMDS) based on generalized UNIFRAC distances [16], analysis of similarity (ANOSIM) and taxonomic binning were performed using Rhea [15] scripts. Distance-based Redundancy Analysis (db-RDA) and constrained correspondence analysis (ANOVA CCA) with 1000 perturbations were performed with the capscale function of the R vegan package. ANOVA CCA p-values were compared to the ANOSIM p-values for the same categories. Serial-group comparisons were performed using Rhea [15]: The default settings were used for the caecum samples as they are set for gut microbiome analysis. The cut-offs were decreased for the lung samples, due to the lower bacterial concentration. To test for significant group separation according to categorical metadata we used PERMANOVA (999 permutations) for all groups. Pairwise categorical differences between taxa and zOTUs were calculated using the Wilcoxon-Rank-Sum-Test with FDH correction. For correlation analysis [15], taxonomic variables were centre log-transformed to remove compositional constraints, and then Pearson correlations within lineages were calculated for all pairs and tested for significance before and after Benjamin-Hochberg (FDH) correction.

Additionally, all other data generated in the study are shown as mean with their respective ranges. Statistical significance for immune data was calculated with GraphPad Prism 6 software (GraphPad Software Inc., San Diego, CA, USA) using 1-way analysis of variance (ANOVA) and Tukey´s multiple comparison as post test, and statistical significance was set to p <0.05.

**Figures and Tables**

**Suppl. Figure 1**

**
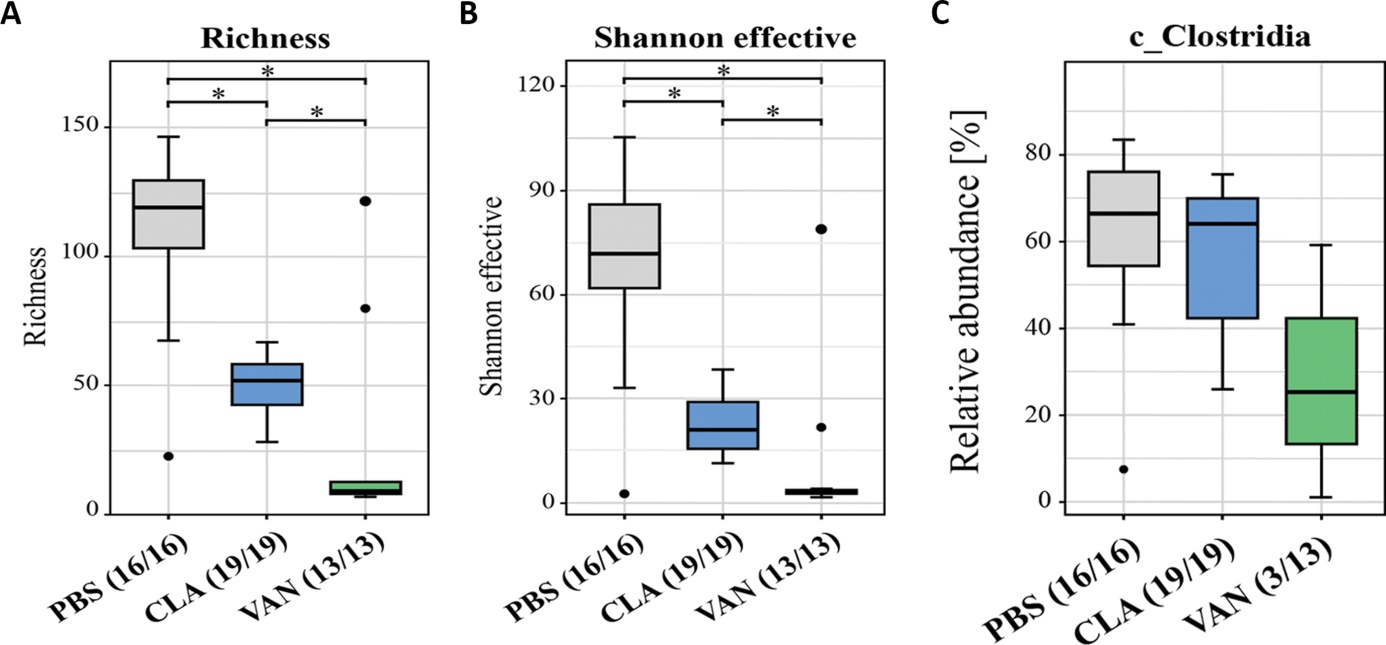
**

**Suppl. Figure 1.** Bacterial analysis of caecal samples: **(A-B)** α-diversity metrics of caecal bacterial species, n=48. **(C)** Impact of antibiotic treatment on the class Clostridia. PBS (n = 16), CLA (n = 19), VAN (n = 13). In the brackets below the graphs e.g. PBS (16/16) in Suppl. Figure 2A means the species was detected in 16 samples out of the overall samples (16) analysed. *p < 0.05

**Suppl. Figure 2**

**
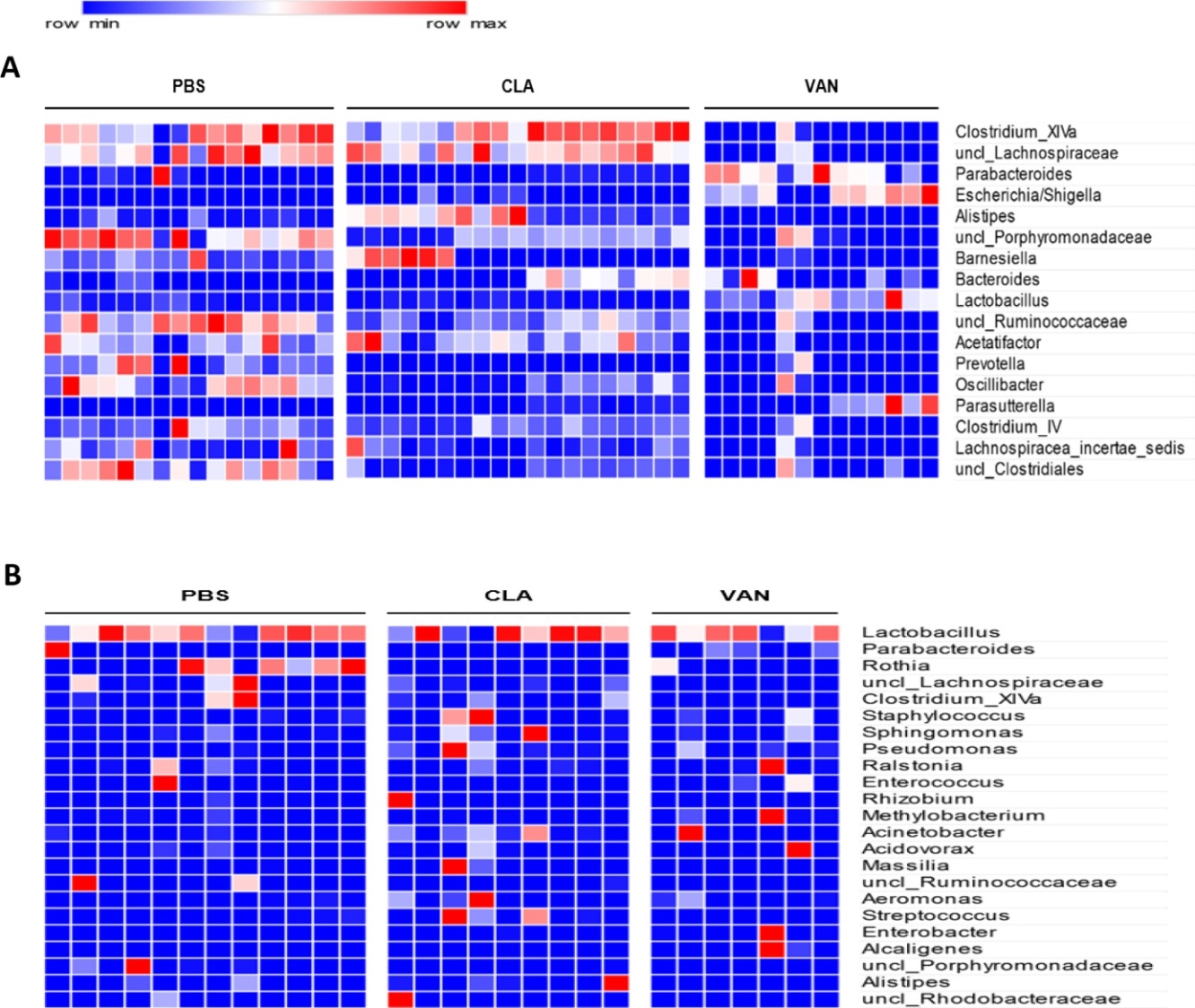
**

**Suppl. Figure 2.** Heat maps showing genera analysed in the caecum and lung: (**A**) Most abundant genera in the caecum. Each accounts for > 0.5% of total relative abundance in all samples. PBS (n = 16), CLA (n = 19), and VAN (n = 13). (**B**) Most abundant genera in the lung. Each accounts for > 0.5% total relative abundance in all samples. PBS (n = 12), CLA (n = 9), and VAN (n = 7), uncl (unclassified).

**Suppl. Table 1**

Twelve most abundant bacterial genera in the caecum.

| **Genus** | **PBS** | **CLA** | **VAN** |
| --- | --- | --- | --- |
| *Clostridium_XlVa* | 25.6 | 27.8 | 2.0 |
| *uncl_Lachnospiraceae* | 19.2 | 20.0 | 2.2 |
| *Parabacteroides* | 6.5 | 1.9 | 36.7 |
| *Escherichia/Shigella* | 0.0 | 2.6 | 32.3 |
| *Alistipes* | 2.8 | 15.8 | 0.1 |
| *uncl_Porphyromonadaceae* | 13.4 | 5.0 | 2.3 |
| *Barnesiella* | 8.0 | 9.6 | 0.6 |
| *Bacteroides* | 0.8 | 6.6 | 5.8 |
| *Lactobacillus* | 1.7 | 0.8 | 11.2 |
| *uncl_Ruminococcaceae* | 4.9 | 1.9 | 0.6 |
| *Acetatifactor* | 2.6 | 2.8 | 0.2 |
| *Prevotella* | 3.7 | - | 0.7 |

Genera are sorted based on overall relative abundance of all samples. Data are presented as mean (%) across samples from each antibiotic treatment and control for ceacal samples. PBS – Control (n = 16), CLA – Clarithromyin (n = 19), VAN – Vancomycin (n = 13), uncl – unclassified.

**Suppl. Figure 3**

**
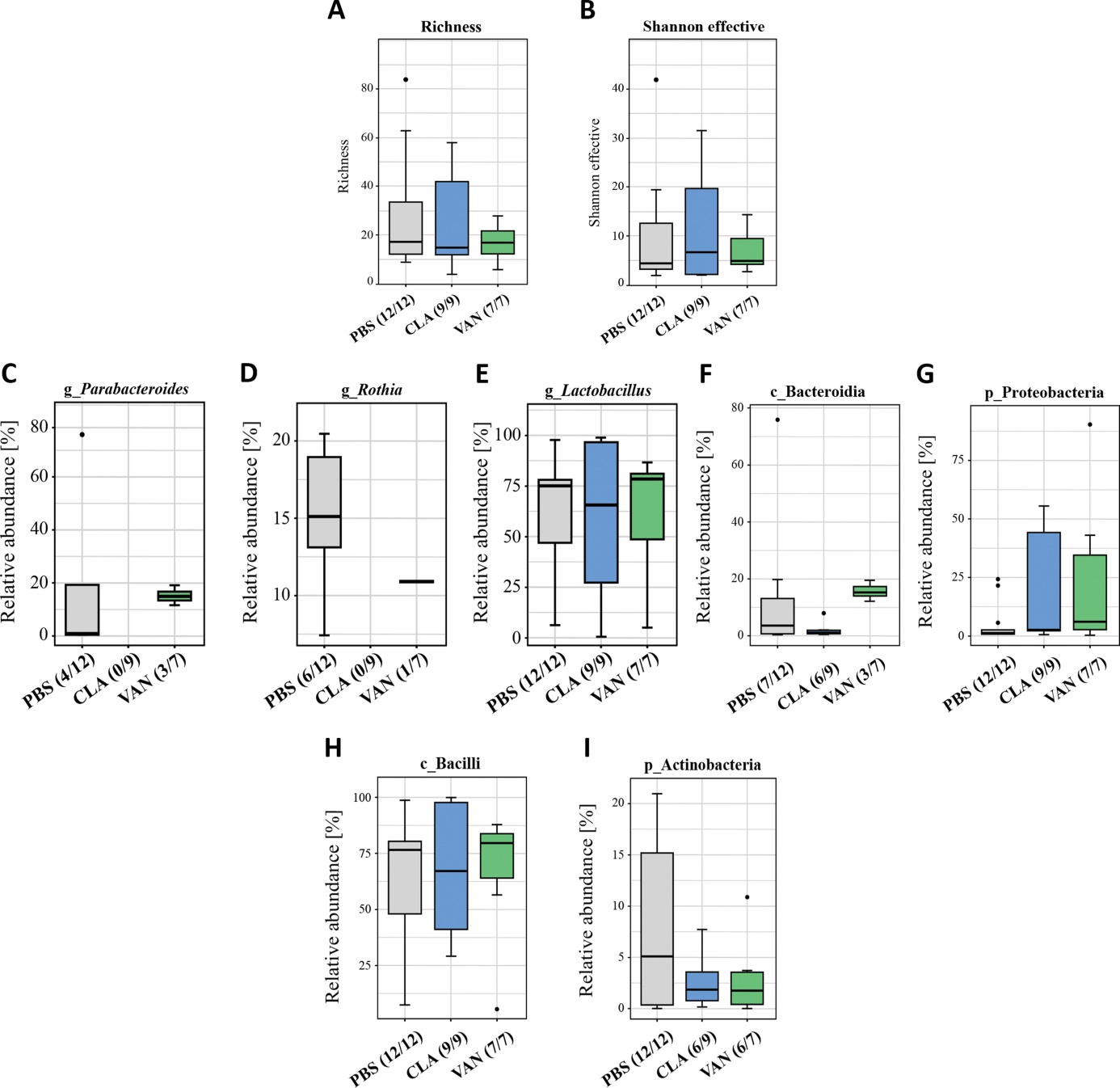
**

**Suppl. Figure 3.** Bacterial analysis of lung samples: **(A-B)** α-diversity metrics of lung bacterial species, n=28. **(C-I)** Impact of antibiotics on some specific lung bacterial taxa. g (genus), p (phylum), c (class). PBS (n = 12), CLA (n = 9), and VAN (n = 7). In the brackets below graphs e.g. CLA (6/9) in Suppl. Figure 4I means the p_Actinobacteria was detected in 6 samples out of the overall samples (9) analysed.

**Suppl. Table 2**

| **Genus** | **PBS** | **CLA** | **VAN** |
| --- | --- | --- | --- |
| *Lactobacillus* | 62.4 | 62.4 | 61.9 |
| *Parabacteroides* | 6.4 | - | 6.5 |
| *Rothia* | 7.6 | - | 1.6 |
| *uncl_Lachnospiraceae* | 5.6 | 1.9 | - |
| *Clostridium_XlVa* | 4.8 | 2.9 | - |
| *Staphylococcus* | 0.3 | 5.2 | 2.3 |
| *Sphingomonas* | 0.4 | 2.7 | 0.9 |
| *Pseudomonas* | 0.1 | 3.0 | 1.2 |
| *Ralstonia* | 1.1 | 0.6 | 2.3 |
| *Enterococcus* | 1.3 | - | 1.5 |

Ten most abundant bacterial genera in the lung.

Genera are sorted based on overall relative abundance of all samples. Data are presented as mean (%) across samples from each antibiotic treatment and control for lung samples. PBS – Control (n = 12), CLA – Clarithromyin (n = 9), VAN – Vancomycin (n = 7), uncl: unclassified.

**Suppl. Figure 4**

**
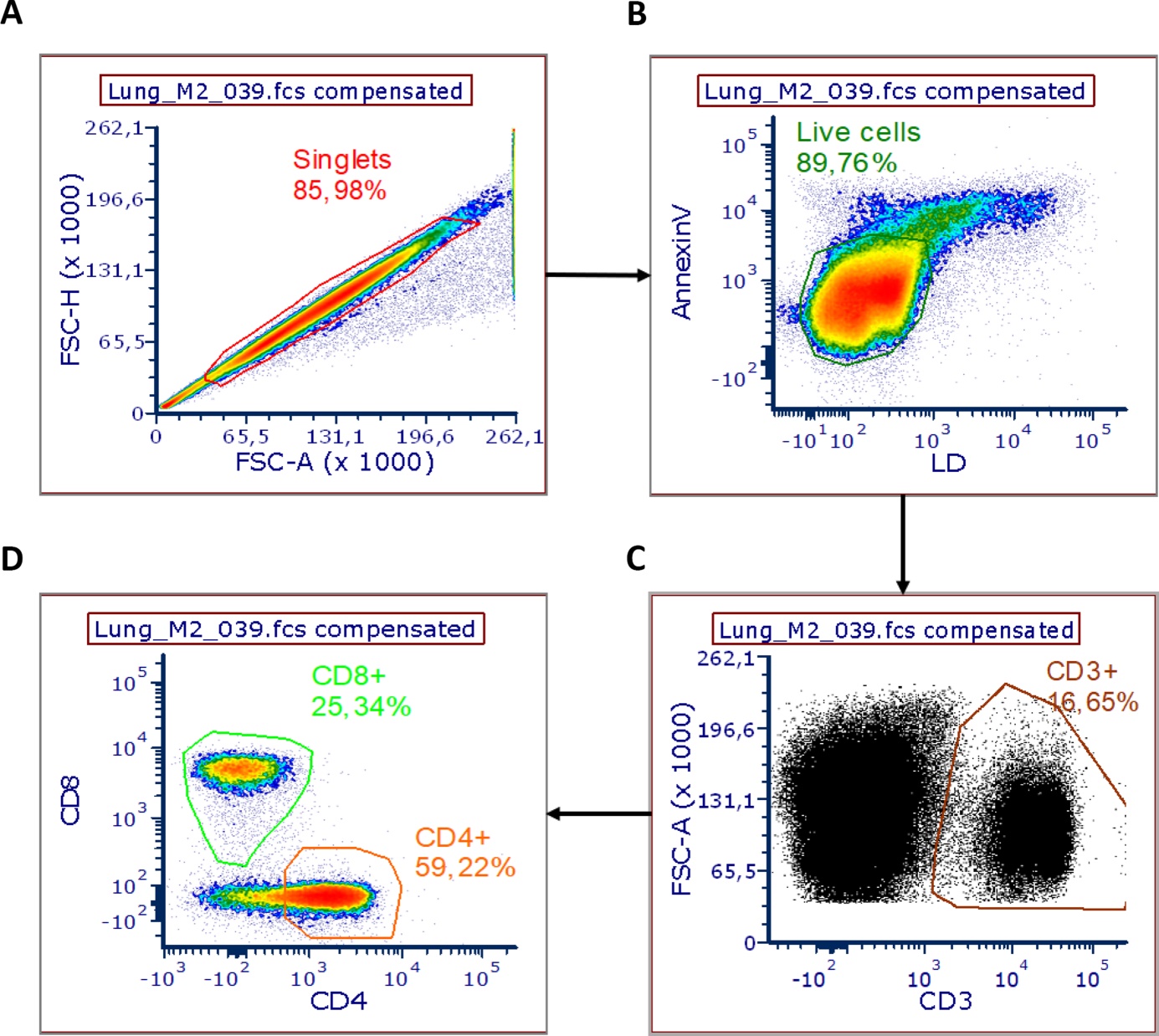
**

**Suppl. Figure 4.** Gating strategy for flow cytometry of the T cell populations in the lungs: **(A-B)** Doublets were excluded and only living cells were gated (double negative for Annexin-V and commercial live-dead dye). **(C)** Lung T cells specified with the marker CD3. **(D)** Classification of the subpopulations of T cells with the markers CD4 and CD8.

**Suppl. Figure 5**

**
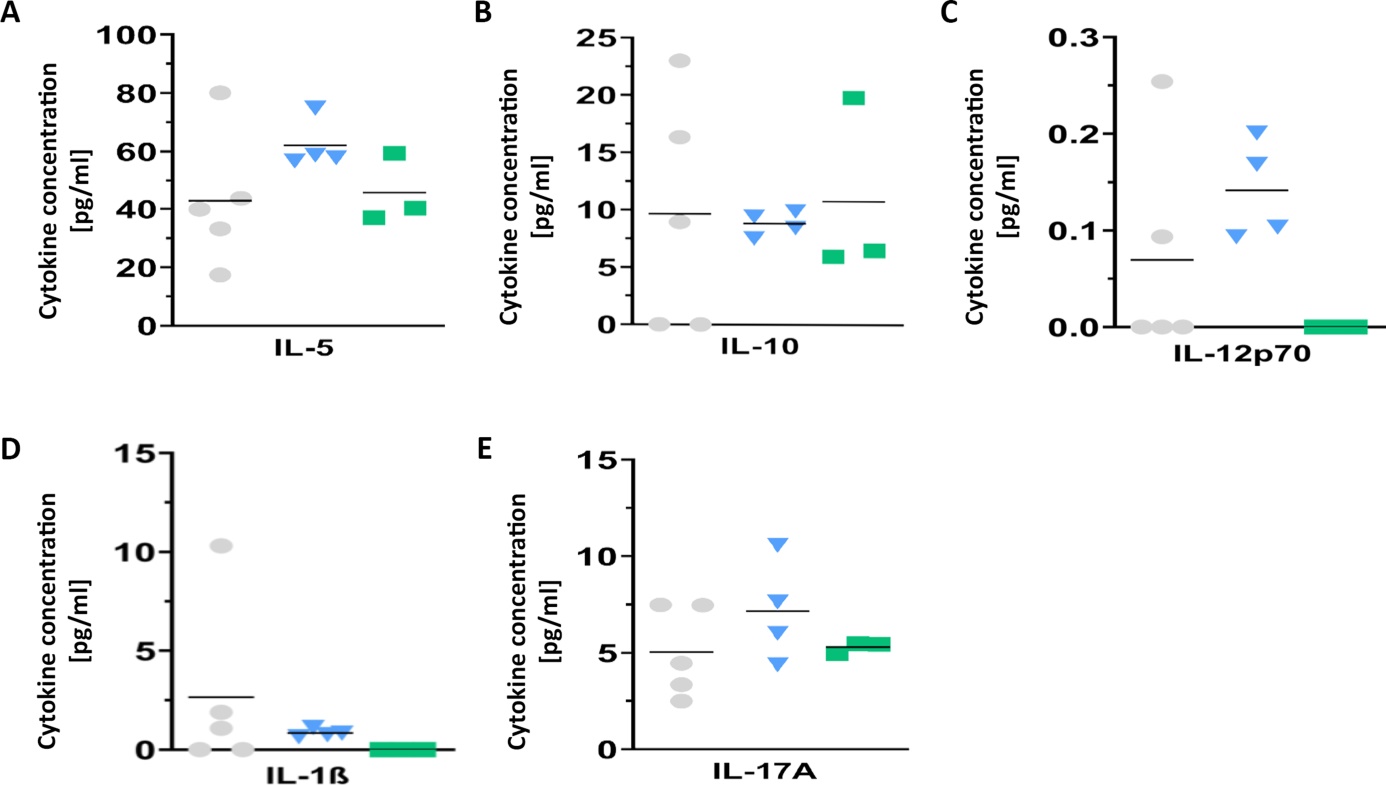
**

**Suppl. Figure 5.** Immune analyses of lung samples: **(A-E)** Cytokines in the supernatant of CD3/CD28 stimulated cell cultures of lung cells. n = 3-5 pooled samples of 2 independent experiments. n for PBS, CLA, and VAN are 5, 4, 3, respectively, and each n represents pooled samples. *p < 0.05 represents significant difference.
